# Stability-indicating HPLC-DAD/UV-ESI/MS impurity profiling of the anti-malarial drug lumefantrine

**DOI:** 10.1186/1475-2875-10-51

**Published:** 2011-02-28

**Authors:** Mathieu Verbeken, Sultan Suleman, Bram Baert, Elien Vangheluwe, Sylvia Van Dorpe, Christian Burvenich, Luc Duchateau, Frans H Jansen, Bart De Spiegeleer

**Affiliations:** 1 Drug Quality and Registration (DruQuaR) group, Faculty of Pharmaceutical Sciences, Ghent University, Harelbekestraat 72, B-9000 Ghent, Belgium; 2 Department of Physiology and Biometrics, Faculty of Veterinary Medicine, Ghent University, Salisburylaan 133, B-9820 Merelbeke, Belgium; 3 School of Pharmacy, Jimma University, Jimma, Ethiopia; 4 Foundation ACT-ion Afrique, B-1000 Brussel, Belgium

## Abstract

**Background:**

Lumefantrine (benflumetol) is a fluorene derivative belonging to the aryl amino alcohol class of anti-malarial drugs and is commercially available in fixed combination products with β-artemether. Impurity characterization of such drugs, which are widely consumed in tropical countries for malaria control programmes, is of paramount importance. However, until now, no exhaustive impurity profile of lumefantrine has been established, encompassing process-related and degradation impurities in active pharmaceutical ingredients (APIs) and finished pharmaceutical products (FPPs).

**Methods:**

Using HPLC-DAD/UV-ESI/ion trap/MS, a comprehensive impurity profile was established based upon analysis of market samples as well as stress, accelerated and long-term stability results. *In-silico *toxicological predictions for these lumefantrine related impurities were made using Toxtree^® ^and Derek^®^.

**Results:**

Several new impurities are identified, of which the desbenzylketo derivative (DBK) is proposed as a new specified degradant. DBK and the remaining unspecified lumefantrine related impurities are predicted, using Toxtree^® ^and Derek^®^, to have a toxicity risk comparable to the toxicity risk of the API lumefantrine itself.

**Conclusions:**

From unstressed, stressed and accelerated stability samples of lumefantrine API and FPPs, nine compounds were detected and characterized to be lumefantrine related impurities. One new lumefantrine related compound, DBK, was identified and characterized as a specified degradation impurity of lumefantrine in real market samples (FPPs). The *in-silico *toxicological investigation (Toxtree^® ^and Derek^®^) indicated overall a toxicity risk for lumefantrine related impurities comparable to that of the API lumefantrine itself.

## Background

**Figure 1:**
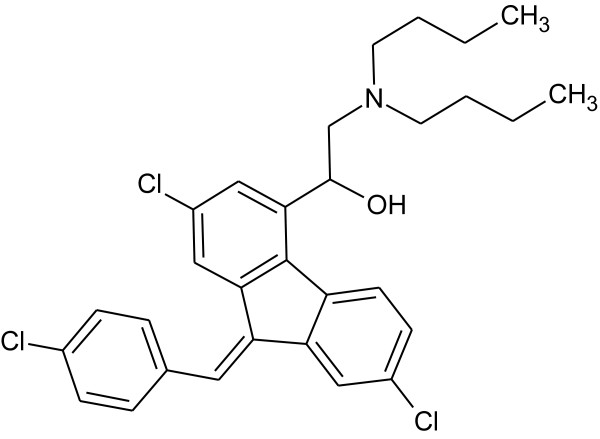
**Structure of Lumefantrine**.

Lumefantrine (benflumetol) is a 2,4,7,9-substituted fluorene (2,3-benzindene) derivative (Figure 1). It was synthesized in the 1970s by the Academy of Military Medical Sciences, in Beijing, and registered in China for anti-malarial use in 1987. It is now commercially available in fixed combination products, mostly with β-artemether (ACT, artemisinin-based combination therapy), which are proven to be highly efficacious for treatment of uncomplicated *falciparum *malaria. In addition to the compound itself, the compound proved to possess marked blood schizontocidal activity against a wide range of *Plasmodium*, among them chloroquine-resistant *Plasmodium falciparum *[1-5].

Biochemical studies suggest that its anti-malarial effect involves lysosomal trapping of the drug in the food vacuole of the intra-erythrocytic parasite, followed by binding to haem that is produced in the course of haemoglobin digestion. This binding prevents the polymerization of haem into haemozoin, hence inhibiting the detoxification of haem. Investigations involving aryl-methanol compounds have suggested the coordination of the iron centre of haem (Fe(III)PPIX) and related porphyrins by the alcohol functionality, indicating the structural activity relationship of the anti-malarial drug lumefantrine [6]. Hence, structural analogues of lumefantrine also posses marked anti-malarial effects. Halofantrine, an aryl amino alcohol analogue of lumefantrine, is also an anti-malarial drug, but is known to be potentially cardiotoxic [7]. Monodesbutyl-benflumetol, a metabolite of lumefantrine, exerts higher blood schizontocidal activity in *Plasmodium falciparum*, as well as in *Plasmodium vivax*. It is about 10-times more effective then lumefantrine [8]. The secondary alcohol permits the formation of dextrorotatory and levorotatory lumefantrine enantiomers and routine synthesis yield the racemate of (+)-lumefantrine and (-)-lumefantrine, which have almost identical potency. Therefore, from the activity point of view, there is no reason to use only one of the enantiomers of lumefantrine instead of the racemate. Moreover, in view of the low animal and human toxicity of the lumefantrine racemate, no major toxicological differences between the two enantiomers are expected [9]. However, other impurities resulting from synthesis might be present.

Lumefantrine containing combinations are incorporated in the WHO essential drug list for the treatment of malaria in endemic areas of the tropical climate. Due to the logistic system [10], degradation products may be spontaneously generated during distribution and storage. Control of such impurities in drug substances and finished drug products is required as they might impart different efficacy and bioavailability to the drug and/or they might produce different adverse and toxic effects to the patients [11].

The safety of a drug product is dependent not only on the toxicological properties of the active drug substance, but also on the toxicological properties of its impurities [12]. Thus, there is an ever-increasing interest in impurities present in APIs and FPPs [13]. Impurity profiling (*i.e. *the identity as well as the quantity of impurities in the pharmaceutical drug) is now gaining critical attention from regulatory authorities. The different Pharmacopoeias, such as the European Pharmacopoeia (Ph.Eur.), United States Pharmacopeia (USP) and International Pharmacopoeia (Ph.Int.) are incorporating specification limits to acceptable levels of impurities present in the API's or FPPs formulations, based upon found levels in approved market samples [11,14,15]. Moreover, ICH guideline Q3A(R) stipulates different thresholds or action limits based upon the maximum daily dose (MDD). For lumefantrine formulations (FPP), with a MDD of 960 mg/day, these are defined as 0.10% reporting threshold, 0.20% identification threshold and 0.20% qualification threshold [16].

USP Salmous (Standards for Articles Legally Marketed Outside the US) and Ph. Int. have already established specification limits for three lumefantrine related impurities: lumefantrine related compound A ((*RS,Z*)-2-(Dibutylamino)-2-(2,7-dichloro-9-(4-chlorobenzylidene)-9*H*-fluoren-4-yl)ethanol), lumefantrine related compound B_A _((1S,3R,5R)-1,3-bis((EZ)-2,7-Dichloro-9-(4-chlorobenzylidene)-9H-fluoren-4-yl)-2,6-dioxabicyclo[3.1.0]hexane) and lumefantrine related compound B_B _((2-((EZ)-2,6-Dichloro-9-(4-chlorobenzylidene)-9H-fluoren-4-yl)-3'-((EZ)-2,7-dichloro-9-(4-chlorobenzylidene)-9H-fluoren-4-yl)-2,2'-bioxirane). The USP Salmous specification limits of these impurities are 0.1% for both impurities A and B_A _and 0.3% for impurity B_B _[17]. The Ph.Int. lumefantrine monograph lists the same three compounds as identified potential impurities, with specification limits of 0.1% for impurity A and 0.3% for impurity B_A _and B_B _[18].

Analytical procedures have been reported for the assay of lumefantrine in different FPPs, using HPLC-UV [19-22]. Furthermore, an LC/MS/MS bio-analytical method for quantification of lumefantrine in human plasma has been developed [23]. However, no impurity profile has been established for this drug, while this is considered much more critical than the assay value. In this study, the potential impurities are described, including new degradants, as well as their relevance towards specification settings and *in-silico *toxicological evaluation. APIs and FPPs containing lumefantrine were evaluated by HPLC, with UV detection for quantification and with ESI-iontrap MS detection for identification.

## Methods

### Samples and chemicals

All drug substance batches (APIs), commercially available FPPs (Co-Artesiane^®^, Artefan^®^, Lumartem^® ^and Coartem^®^) and the standard of desbenzylketo (DBK) lumefantrine derivative were supplied by Dafra Pharma International (Belgium). USP-Salmous standards of lumefantrine and impurity A were purchased from U.S. Pharmacopeia (Basel, Switzerland). Analytical solutions were prepared in HPLC grade tetrahydrofuran (Fisher Scientific, Leicestershire, UK) at a concentration of 0.96 mg/ml lumefantrine, which corresponds to 100% label claim (l.c.). A dilution equivalent to 0.5% l.c. is also prepared and used for the quantification of the related impurities. Hydrogen peroxide (H_2_O_2_), sodium hydroxide (NaOH) and ammonium acetate were purchased from Merck (Darmstadt, Germany), hydrochloric acid (HCl) from Sigma-Aldrich (St Louis, USA) and glacial acetic acid from Riedel-de Haën (Seelze, Germany). Sartorius (Göttingen, Germany) ultrapure 18.2 MΩ.cm quality water and HPLC grade acetonitril (Romil, Cambridge, UK) were used for HPLC-UV/MS analysis.

### Liquid chromatography

HPLC-UV investigation of the impurity profiles was performed on a HPLC-PDA apparatus consisting of a Waters Alliance 2695 separation module and a Waters 2998 photodiode array detector with Empower 2 software for data acquisition (all Waters, Milford, MA, USA). For PDA detection, the UV spectrum was recorded at 190-400 nm. Quantification was performed at 266 nm. The positive ion ESI and the collision-induced dissociation (CID) mass spectra were obtained from the LC-UV/MS apparatus consisting of a Spectra System SN4000 interface, a Spectra System SCM1000 degasser, a Spectra System P1000XR pump, a Spectra System AS3000 autosampler, and a Finnigan LCQ Classic ion trap mass spectrometer in positive ion mode (all Thermo, San José, CA, USA), mass to charge range m/z 100 to m/z 2000 at unit resolution and with a peak width of 0.25 daltons/z, equipped with a Waters 2487 dual wavelength UV detector (Waters, Milford, MA, USA) and Xcalibur 2.0 software (Thermo) for data acquisition. ESI was conducted using a needle voltage of 4.5 kV. Nitrogen was used as sheath and auxiliary gas with the heated capillary set at 250°C. UV-detection was used for quantification (at 266 nm), while ESI-ion trap MS detection was used for identification.

LC determination of impurities in lumefantrine samples was performed using a Purospher STAR RP-18 endcapped (150 × 4.6 mm, 5 μm particle size) column (Merck, Darmstadt, Germany) with guard column at 30°C under isocratic conditions with a mobile phase consisting of ammonium acetate (pH 4.9; 0.1 M) and acetonitrile (10:90, v/v). The flow rate was set at 2.0 mL/min (minimal run time: 30 min.). The injection volume was 10 μl. Under these conditions, lumefantrine elutes at approximately 22 min. System suitability tests (SSTs) were established as the plate number on lumefantrine (N ≥ 8.2 × 10^3^) and the oxidative stress degradation product (N ≥ 2.4 × 10^3^), the signal-to-noise ratio of 0.5% l.c. lumefantrine solution (S/N ≥ 30), the peak area ratio of the 0.5% l.c. versus 100% l.c. (between 0.4 and 0.6) and the relative position of the *in-situ *prepared N-oxide by H_2_O_2 _treatment (RRT between 0.12 and 0.22).

The LC method was validated for the determination of lumefantrine and its related impurities. The selectivity of the developed chromatographic method was established by the separation of lumefantrine and its impurities. A correlation coefficient (r^2^) of 0.9998 for lumefantrine (0.0006 to 0.01 mg/ml) and 1.0 for impurity A and DBK (0.001 to 0.1 mg/ml) demonstrated that the HPLC method is linear in the lower range. LOD/LOQ values for lumefantrine, DBK and impurity A were calculated (S/N = 3 resp. 10): 0.004 mg/ml and 0.026 mg/ml for lumefantrine (0.004% respectively 0.026% l.c.), 0.011 mg/ml and 0.040 mg/ml for DBK (0.012% respectively 0.042% l.c.) and 0.110 mg/ml and 0.393 mg/ml for impurity A (0.115% respectively 0.409% l.c.). The analytical stability of lumefantrine, impurity A and DBK was confirmed over a storage period of 24 hours at 5°C, *i.e. *the sample compartment temperature. Accuracy and precision were evaluated by repeated analysis (n = 6), with 102.6% l.c. recovery and 2.1%, respectively 2.86%, for repeatability, respectively intermediate precision.

The relative retention time (RRT) is defined as the ratio of the retention time of the compound versus the retention time of lumefantrine. The relative response factor is defined as the ratio of the area of the compound versus the area of lumefantrine, both injected at the same concentration.

### Forced degradation

Forced degradation of lumefantrine API and FPP was performed under heat, light, acidic, alkaline and oxidative stress conditions. In heat stress studies, the FPP powder (one gram) was incubated at 40, 50 and 60°C for respectively four, three and two days. The placebo powder (one gram) was incubated for two days at 60°C. In light stress studies, the FPP and placebo powder (one gram) were subjected to UV (three days incubation) and VIS (seven days incubation) light in a qualified Pharma 500 L stability cabinet (Weiss Technik) according to ICH. Finally, FPP and placebo were stressed by adding 10 ml of 1 M HCl (acidic), 1 M NaOH (alkaline) or 1% H_2_O_2 _(oxidative) to one gram of the powder to be examined. Samples were incubated, up to eight days, at 5, 25, 40, 50 and 60°C. After the incubation, samples were neutralized using NaOH (acidic), HCl (alkaline) or Na_2_S_2_O_5 _(oxidative), and the solvent evaporated using freeze-drying. The resulting powdered samples were dispersed in THF, centrifuged, HPLC-filtrated and analyzed using HPLC-DAD/UV-ESI/MS.

Additionally, different batches of FPP were included in long-term (up to 24 months, 30°C, 70-75% RH) and accelerated (up to 6 months, 40°C, 75% RH) stability studies according to ICH stability guidelines [24].

### *In-silico *toxicological predictions

To make *in-silico *toxicological predictions for lumefantrine and its identified related impurities, two sources of toxicological predictions were used: Derek^® ^(Nexus v2.0) for Windows developed by Lhasa Limited (Leeds, UK) and Toxtree^® ^(v1.60) developed by Ideaconsult Ltd. (Sofia, Bulgaria). Derek^® ^(Nexus v2.0) for Windows is an expert knowledge base system, containing descriptions of molecular substructures which have been associated with toxic endpoints (structural alerts), that predicts whether a chemical is toxic in humans, other mammals and bacteria. The programme applies structure-activity relationships ((Q)SARs) and other expert knowledge rules to derive a reasoned conclusion about the potential toxicity of the query chemical [25,26]. Toxtree^® ^is an open source application, which is able to estimate toxic hazards by applying a decision tree approach and making structure-based predictions for a number of toxicological endpoints using different modules. Hazard estimations were generated using three Toxtree^® ^modules: Cramer rules with extensions, Benigni/Bossa rulebase and structure alerts for the *in vivo *micronucleus assay in rodents.

## Results and discussion

### HPLC analysis of lumefantrine containing samples

As the Ph.Int./USP Salmous HPLC methods [17,18] are a complex, step-wise gradient using an ion-pairing reagent, this method is not compatible with MS detection. Moreover, the gradient is required for the detection of synthesis impurities B_A _and B_B_, which are structurally very different from lumefantrine and its other impurities, especially degradants. Therefore, an isocratic RP-HPLC method was used without an MS-incompatible ion-pairing reagent. The chromatographic characteristics of these two synthesis impurities on this system could not be evaluated, due to the unavailability of references for these impurities.

**Figure 2:**
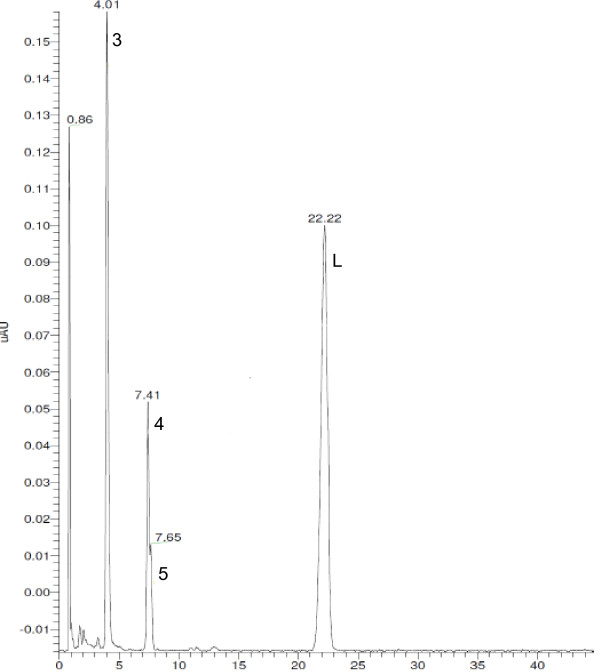
**UV chromatogram of a mixed sample illustrating lumefantrine N-oxide, lumefantrine DBK and desbenzyl derivative and lumefantrine**. UV chromatogram of a mixed sample illustrating lumefantrine N-oxide (3), DBK (4) and desbenzyl lumefantrine derivative (5) and lumefantrine (L).

Lumefantrine API and FPPs were exposed to diverse stress conditions for different periods. Additionally, FPPs were put on long-term and accelerated stability studies as well according to ICH. FPP samples in the long-term stability study were kept for up to twenty-four months at 30°C/75% RH. In the accelerated study, the stability conditions were adjusted and FPP samples were kept for up to six months at 40°C/75% RH. The unstressed and stressed API samples, as well as the unstressed (release), accelerated, long-term and stressed FPP samples were analyzed with the validated HPLC method. Five synthesis and four stress related lumefantrine impurities have been observed in lumefantrine containing samples (Table 1). The relative retention time (RRT), relative to lumefantrine, of these impurities was defined and normalized quantification was performed with a reporting threshold of 0.10%. Maximal actually observed levels of lumefantrine related impurities in different samples under different conditions were obtained (Table 2). None of these lumefantrine related impurities were observed above the reporting threshold (*i.e. *> 0.10%) in unstressed API and release (T_0_) FPP samples, except for the monodesbutyl derivative. However, these lumefantrine degradants were observed in stressed API and FPP samples, and in FPP stability studies. Compounds 1,2 and 3 were observed in oxidative stressed API samples. Three lumefantrine related impurities were observed in stressed FPP stability samples: compound 1 (60°C, 1 M NaOH, T_2d_), compound 3 (60°C, 1% H_2_O_2_, T_2d_) and compound 4 (50°C, 1 M HCl, T_3d_). Compound 3 and 4 were also detected in the accelerated (40°C/75% RH, T_6m_) and long-term stability studies. A typical UV chromatogram illustrating the separation of lumefantrine N-oxide, DBK, desbenzyl lumefantrine derivative and lumefantrine is given in Figure 2.

**Table 1:** Structural information for the observed and/or reported lumefantrine related impurities

#	Compound [formula, mono-isotopic mass]	Structure	Origin
1	Desbenzylketo N-oxide [C_23_H_27_NO_3_Cl_2_, MW 435.14]		Alkaline stress Oxidative stress
2	Lumefantrine (mono-)desbutyl derivative [C_26_H_24_NOCl_3_, MW 473.09]		Oxidative stress Metabolite
3	Lumefantrine N-oxide [C_30_H_32_NO_2_Cl_3_, MW 543.15]		Oxidative stress Degradation
4	2,7-dichloro-4-[2-(di-n-butylamino)-1-hydroxyethyl]-9H-fluoren-9-one; Desbenzylketo derivative (DBK) [C_23_H_27_NO_2_Cl_2_, MW 419.14]		Oxidative stress Acidic stress Degradation
5	2-(di-n-butylamino)-1-[2,7-dichloro-9H-fluoren-4-yl]ethanol; Desbenzyl derivative [C_23_H_29_NOCl_2_, 405.16]		Synthesis
6	Synthesis impurity found in lumefantrine API; Lumefantrine oxide [C_30_H_32_NO_2_Cl_3_, MW 543.14]		Synthesis
7	Synthesis impurity found in lumefantrine API; Lumefantrine oxide [C_30_H_32_NO_2_Cl_3_, MW 543.14]		Synthesis
8	(RS,Z)-2-(Dibutylamino)-2-(2,7-dichloro-9-(4-chloro-benzylidene)-9H-fluoren-4-yl)ethanol (isomeric compound); Impurity A (Ph. Int./USP Salmous) [C_30_H_32_NOCl_3_, MW 527.15]		Synthesis
9	Synthesis impurity found in lumefantrine API; Lumefantrine oxide [C_30_H_32_NO_2_Cl_3_, MW 543.14]		Synthesis
10	(1S,3R,5R)-1,3-bis((EZ)-2,7-Dichloro-9-(4-chlorobenzyl-idene)-9H-fluoren-4-yl)-2,6-dioxabicyclo[3.1.0]hexane; Impurity B_A _(USP Salmous) [C_44_H_24_Cl_6_O_2_, 797.39]		Synthesis
11	2-((EZ)-2,6-Dichloro-9- (4-chlorobenzylidene)-9H-fluoren-4-yl)-3'-((EZ)-2,7-dichloro-9-(4-chlorobenzylidene)-9H-fluoren-4-yl)-2,2'-bioxirane; Impurity BB (USP Salmous) [C_44_H_24_Cl_6_O_2_, 797.39]		Synthesis

**Table 2:** Percentage maximum actual levels of lumefantrine related impurities observed^(1)^

#	Compound	API		FPP		
		Unstressed	Stressed	Release	Accelerated	Stressed
1	Desbenzylketo N-oxide		1.39			0.60
2	Monodesbutyl derivative		0.56	0.11		
3	Lumefantrine N-oxide		21.32		0.12	0.86
4	Desbenzylketo derivative				0.34	4.26
5	Desbenzyl derivative					
6	Lumefantrine oxide (RRT ~ 0.49)					
7	Lumefantrine oxide (RRT ~ 0.52)					
8	Impurity A					
9	Lumefantrine oxide (RRT ~ 0.59)					

(1) RT: reporting threshold = 0.10%

### Identification of lumefantrine impurities with LC-MS/MS

The observed lumefantrine impurity peaks (related to synthesis as well as degradation processes) in stressed or unstressed API and FPPs were identified using LC-MS/MS, with one of them investigated for the first time and proposed as a new specified lumefantrine related impurity. The desbutyl, desbenzyl and isomeric compound A derivatives are already known lumefantrine impurities. The analytical characteristics of the remaining unidentified lumefantrine related impurities were obtained by analysis of MS data: *m/z *values (Table 3), isotopic-distributions in mass spectra (Figure 2) and MS/MS (fragmentation pattern for structural identification).

**Table 3:** HPLC characteristics of lumefantrine related impurities

#	Compound	**RT**^**(1)**^	**RRT**^**(2)**^	Most abundant *m/z *observed
1	Desbenzylketo N-oxide	1.79	0.08	436.14
2	Monodesbutyl derivative	3.25	0.15	474.00
3	Lumefantrine N-oxide	3.96	0.17	544.08
4	Desbenzylketo derivative	7.41	0.33	420.13
5	Desbenzyl derivative	7.69	0.34	406.09
6	Lumefantrine oxide	10.96	0.49	544.12
7	Lumefantrine oxide	11.45	0.52	544.12
8	Impurity A	12.70	0.58	528.10
9	Lumefantrine oxide	12.97	0.59	544.12
L	Lumefantrine	22.28	1.00	528.10

(1) Retention time (min.)

(2) Relative retention time

**Figure 3:**
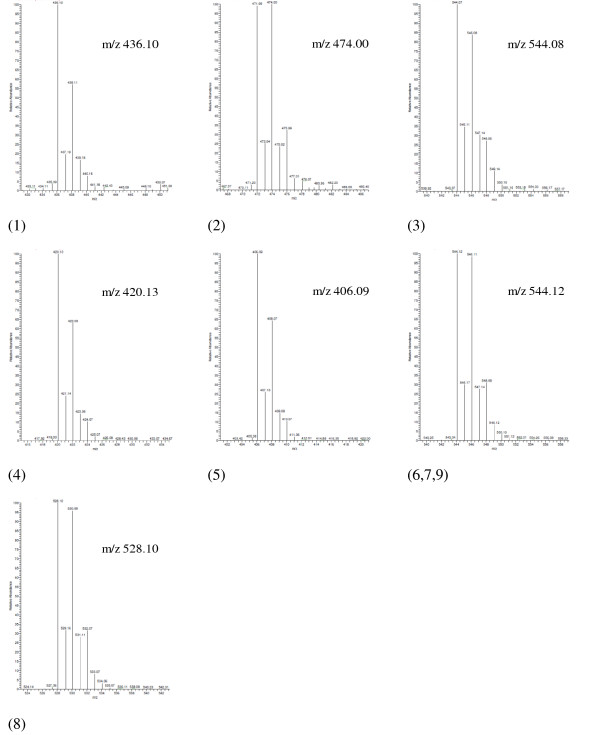
**Isotopic-distribution mass spectra of lumefantrine related impurities with the most abundant m/z observed**. Isotopic-distribution mass spectra of lumefantrine related impurities: (1) Desbenzylketo N-oxide; (2) Monodesbutyl derivative; (3) Lumefantrine N-oxide; (4) Desbenzylketo derivative; (5) Desbenzyl derivative; (6,7,9) Oxide of lumefantrine and (8) Impurity A (USP/Ph.Int).

The mono-isotopic mass of lumefantrine [(1*RS*)-2-(Dibutylamino)-1-[(*Z*)-2,7-dichloro-9-(4-chlorobenzylidene)-9*H*-fluoren-4-yl]ethanol] was calculated to be 527.15. The mass spectrum of lumefantrine main peak indicated the most abundant ion at an *m/z*-ratio of 528.10, with an isotopic distribution corresponding to the three chlorine atoms in its structure (^35^Cl at 75.77% and ^37^Cl at 24.23%). In the mass spectrum of compound 1 (RRT ~ 0.08), 436.14 is observed to be the most abundant *m/z*. The isotopic distribution is suggestive for a compound possessing two chlorine atoms, and is identical to the isotopic distribution of compound 4. The most abundant *m/z *value for compound 4 is 420.13, with a molecular formula of C_23_H_27_NO_2_Cl_2_, *i.e. *desbenzylketo derivative (DBK). This MS-derived structure was confirmed by chemical synthesis of a DBK reference and its IR and NMR spectroscopic structure confirmation. This DBK reference standard gave similar chromatographic retention characteristics as well as DAD-UV spectrum as degradant 4 found in the samples. Based on the observed *m/z *values of compound 1 and DBK, compound 1 has an additional oxide to its structure, and is thus assigned as being the N-oxide of DBK. The most abundant ion found for compound 2 was *m/z *474.00. Its isotopic distribution is characteristic for a compound possessing three chlorines and the molecular formula C_26_H_24_NOCl_3_, *i.e. *the monodesbutyl derivative. As this compound is more hydrophilic than lumefantrine, it elutes much earlier than lumefantrine. Compound 3 was found in the oxidative stressed FPP samples. Its most abundant *m/z *is 544.08, with an isotopic distribution corresponding to that of lumefantrine, giving the molecular formula C_30_H_24_NO_2_Cl_3_. Based on the observed *m/z *values of compound 3 and lumefantrine, compound 3 has an additional oxide to its structure. MS/MS fragmentation spectra, by collision induced dissociation (CID, energy 100 eV), of compound 3 showed peaks at *m/z *526.12 (loss of H_2_O), 470.10, 396.99, 380.95, 346.23, 305.58, 298.06 (loss of C_14_H_7_Cl_2_) and 152.30. This impurity was thus identified as lumefantrine N-oxide. Compounds 6,7 and 9 gave identical mass spectra (Figure 3) with the most abundant ion found at *m/z *544.12. The isotopic distributions are characteristic for a compound containing three chlorines and a molecular formula C_30_H_32_NO_2_Cl_3_, *i.e. *oxides of lumefantrine. As these three impurities are eluting at different retention time, they are most probably isomeric compounds with an -OH function at different positions on the lumefantrine aromatic ring structure.

### Specified lumefantrine impurity DBK

**Figure 4:**
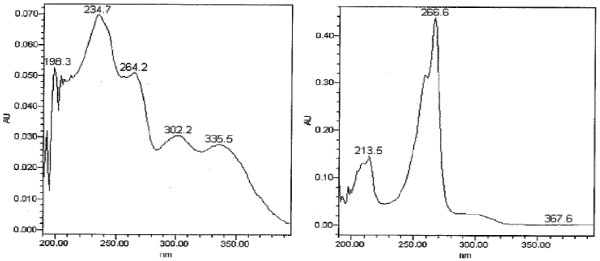
**UV spectra recorded for lumefantrine (left) and DBK (right) showing the observed wavelength of maximum absorption**. DAD-UV spectra of lumefantrine (left) and DBK (right).

The lumefantrine-related compound 4, DBK (RRT ~ 0.33), was not only formed in stress stability samples, but was also observed in accelerated and stressed stability samples of FPP. Moreover, DBK was found to be present in market samples at a concentration ranging between 0.03% and 0.12%, determined by area normalization. Subsequently, DBK was synthesized for further analytical characterization, including confirmation of its relative retention time (RRT) and determination of its relative response factor (RFF) at the detection wavelength of 266 nm. The RRT and the RRF of DBK relative to lumefantrine were experimentally determined to be 0.33 and 2.87 respectively. The DAD-UV spectra recorded for lumefantrine and DBK (Figure 4) showed the wavelength of maximum absorption to be higher for DBK (app. 266 nm) than for lumefantrine (app. 234 nm), due to the benzyl group being replaced by the keto function. This impurity was observed in oxidative and acidic stress degradation, as well as in the accelerated and long-term ICH stability studies, justifying this degradant to be classified as a specified degradant.

### *In-silico *toxicological predictions of lumefantrine and its related impurities

Using the knowledge-based expert systems Toxtree^® ^and Derek^®^, general toxicological and carcinogenic alerts for lumefantrine, as well as for its related observed and already described impurities, have been investigated. Since DBK is a specified lumefantrine-related compound, the toxicity profile of DBK is of paramount importance. Based on the Cramer rules with extensions, Toxtree^® ^clearly predicted general toxicity risks (class III), and genotoxic alerts (polycyclic aromatic hydrocarbons, halogenated benzene and H-acceptor-path3-H-acceptor) for DBK, which are identical to the API lumefantrine itself. According to the toxicological concern (TTC), the daily dosage for compounds classified in class III should be below 90 μg/person (60 kg)/day to be validated as non toxic [27]. Hence, the TTC value of 90 μg on the MDD of 960 mg lumefantrine corresponds to a limit of 0.01% (90 μg/960 mg), which is far below the levels actually observed.

The toxicity profile by Derek^® ^of DBK is defined by several general toxicity alerts which are similar to lumefantrine: hERG channel inhibion and α_2 μ_-globulin nephropathy [28] plus additional photo-toxicity and -allergenicity. However, Derek^® ^did not trigger any genotoxicity or carcinogenicity for DBK.

The other lumefantrine related impurities were also predicted in Toxtree^® ^to have a high general toxicity similar to lumefantrine itself (depicted Class III), based on the Cramer rules with extensions, and genotoxicity risks. Again, Derek^® ^clearly indicated a limit toxicity profile for the majority of lumefantrine related impurities compared to lumefantrine (hERG channel inhibion, α_2μ_-globulin nephropathy). Only impurity B_B _triggered additional toxicity alerts (carcinogenicity/mutagenicity, chromosome damage, eye/skin irritation, developmental toxicity, skin sensitization), indicative for a non-toxic profile compared to lumefantrine itself.

## Conclusions

An exhaustive impurity profiling of lumefantrine was performed using HPLC-UV/ESI-ion trap MS. From unstressed, stressed and accelerated stability samples of lumefantrine API and FPPs, nine compounds were detected and characterized to be lumefantrine related impurities. One new lumefantrine related compound, DBK, was identified and characterized as a specified degradation impurity of lumefantrine in real market samples (FPPs). The *in-silico *toxicological investigation (Toxtree^® ^and Derek^®^) indicated overall a lesser toxicity for the specified impurity DBK compared to the API lumefantrine itself.

## Competing interests

The authors, MV, BB, EVG and BDS would like to acknowledge that Dafra sponsored the analytical development. However, these authors do not work for, or represent in any way, Dafra. FHJ is partly working for Dafra.

## Authors' contributions

MV and SS did part of the analytical experiments incl. validation, performed the *in-silico *verification and wrote the article. BB, EVG and SVD did part of the analytical experiments, incl. stability and MS experiments and QC on data. CB and LD critically reviewed and discussed this manuscript. FHJ designed the experiments, overviewed the DBK reference synthesis, and critically reviewed the manuscript. BDS was the overall study director, responsible for design of experiments, interpretation of data and writing this manuscript. All authors read and approved the final manuscript.

## References

[B1] Falade C Makanga M Premji Z Ortmann CE Stockmeyer M de Palacios PI Efficacy and safety of artemether-lumefantrine (Coartem (R)) tablets (six-dose regimen) in African infants and children with acute) uncomplicated *falciparum *malaria Trans R Soc Trop Med Hyg 2005 99 459 467 10.1016/j.trstmh.2004.09.013 15837358

[B2] Hatz C Abdulla S Mull R Schellenberg D Gathmann I Kibatala P Beck HP Tanner M Royce C Efficacy and safety of CGP 56697 (artemether and benflumetol) compared with chloroquine to treat acute *falciparum *malaria in Tanzanian children aged 1-5 years Trop Med Int Health 1998 3 498 504 10.1046/j.1365-3156.1998.00250.x 9657513

[B3] van Vugt M Concerns about the privatization of public goods: A social dilemma analysis Soc Psychol Q 1997 60 4 355 367 10.2307/2787095

[B4] van Vugt M Looareesuwan S Wilairatana P McGready R Villegas L Gathmann I Mull R Brockman A White NJ Nosten F Artemether-lumefantrine for the treatment of multidrug-resistant *falciparum *malaria Trans R Soc Trop Med Hyg 2000 94 545 548 10.1016/S0035-9203(00)90082-8 11132386

[B5] vonSeidlein L Jaffar S Pinder M Haywood M Snounou G Gemperli B Gathmann I Royce C Greenwood B Treatment of African children with uncomplicated *falciparum *malaria with a new antimalarial drug, CGP 56697 J Infect Dis 1997 176 1113 1116 10.1086/516524 9333180

[B6] de Villiers KA Egan TJ Recent advances in the discovery of haem-targeting drugs for malaria and schistosomiasis Molecules 2009 14 2868 2887 10.3390/molecules14082868 19701131 PMC6254801

[B7] Traebert M Dumotier B Meister L Hoffmann P Dominguez-Estevez M Suter W Inhibition of hERG K+ currents by antimalarial drugs in stably transfected HEK293 cells Eur J Pharmacol 2004 484 41 48 10.1016/j.ejphar.2003.11.003 14729380

[B8] Pirker-Krassnig DK Wernsdorfer G Sirichaisinthop J Rojanawatsirivet C Kollaritsch H Wernsdorfer WH Comparative study on the *in vitro *activity of lumefantrine and desbutyl-benflumetol in fresh isolates of *Plasmodium vivax *from Thailand Wien Klin Wochenschr 2004 116 47 52 10.1007/BF03040424 15683043

[B9] Wernsdorfer WH Landgraf B Kilimali V Wernsdorfer G Activity of benflumetol and its enantiomers in fresh isolates of *Plasmodium falciparum *from East Africa Acta Trop 1998 70 9 15 10.1016/S0001-706X(97)00141-1 9707360

[B10] Ballereau F Prazuck T Schrive I Lafleuriel MT Rozec D Fisch A Lafaix C Stability of essential drugs in the field: Results of a study conducted over a two-year period in Burkina Faso Am J Trop Med Hyg 1997 57 31 36 9242314 10.4269/ajtmh.1997.57.31

[B11] Bari SBKB Jaiswal YS Shikhedkar AA Impurity profile: Significance in active pharmaceutical ingredient Eurasian J Anal Chem 2007 2 1 32 53

[B12] Vergote V Burvenich C Van de Wiele C De Spiegeleer B Quality specifications for peptide drugs: a regulatory-pharmaceutical approach J Pept Sci 2009 15 697 710 10.1002/psc.1167 19750489

[B13] De Spiegeleer B Vergote V Pezeshki A Peremans K Burvenich C Impurity profiling quality control testing of synthetic peptides using liquid chromatography-photodiode array-fluorescence and liquid chromatography-electrospray ionization-mass spectrometry: The obestatin case Anal Biochem 2008 376 229 234 10.1016/j.ab.2008.02.014 18342612

[B14] Nicolas EC Scholz TH Active drug substance impurity profiling - Part I. LC/UV diode array spectral matching J Pharm Biomed Anal 1998 16 813 824 10.1016/S0731-7085(97)00131-3 9535194

[B15] Roy J Pharmaceutical impurities - A mini review AAPS PharmSciTech 2002 3 article 6 10.1208/pt030206 PMC2750308 12916943

[B16] ICH guidelines - International Conference on Harmonization, Q3A(R2) Impurities in new drug substances CPMP/ICH/2737/99. (October 2006) http://www.ema.europa.eu/docs/en_GB/document_library/Scientific_guideline/2009/09/WC500002675.pdf [Accessed on 5 November 2010 at 11:06]

[B17] Authorized Lumefantrine USP Salmous Standard (Februari 2009) http://www.usp.org/pdf/EN/nonUSStandards/lumefantrine.pdf [Accessed on 24 October 2010 at 17:14]

[B18] Lumefantrine: Document QAS/06.186/FINAL (WHO Ph. Int. - July 2008) http://www.who.int/medicines/publications/pharmacopoeia/Lumef_monoFINALQAS06_186_July08.pdf [Accessed on 24 October 2010 at 17:37]

[B19] Lee H Pharmaceutical applications of liquid chromatography coupled with mass spectrometry (LC/MS) J Liq Chromatogr Relat Technol 2005 28 1161 1202 10.1081/JLC-200053022

[B20] Cesar ID Nogueira FHA Pianetti GA Simultaneous determination of artemether and lumefantrine in fixed dose combination tablets by HPLC with UV detection J Pharm Biomed Anal 2008 48 951 954 10.1016/j.jpba.2008.05.022 18602241

[B21] Cesar ID Nogueira FHA Pianetti GA Comparison of HPLC, UV spectrophotometry and potentiometric titration methods for the determination of lumefantrine in pharmaceutical products J Pharm Biomed Anal 2008 48 223 226 10.1016/j.jpba.2008.05.006 18571353

[B22] Patil KR Rane VP Sangshetti JN Shinde DB A Stability-Indicating LC Method for Lumefantrine Chromatographia 2009 69 375 379 10.1365/s10337-008-0894-x

[B23] Munjal V Paliwal N Chaursia BK Varshney B Ahmed T Paliwal J LC-tandem mass spectrometry method for quantification of lumefantrine in human plasma and its application to bioequivalence study Chromatographia 2010 71 505 510 10.1365/s10337-009-1446-8

[B24] ICH guidelines - International Conference on Harmonization, Q1A(R2) Stability testing of new drug substances and products CPMP/ICH/2736/99. (August 2003) http://www.ema.europa.eu/docs/en_GB/document_library/Scientific_guideline/2009/09/WC500002651.pdf [Accessed on 5 November 2010 at 12:10]

[B25] Ellison CM Madden JC Judson P Cronin MTD Using *in silico *tools in a weight of evidence approach to aid toxicological assessment Mol Inf 2010 29 97 110 10.1002/minf.200900006 27463852

[B26] Mohan CG Gandhi T Garg D Shinde R Computer-assisted methods in chemical toxicity prediction Mini Rev Med Chem 2007 7 499 507 10.2174/138955707780619554 17504185

[B27] Munro IC Renwick AG Danielewska-Nikiel B The threshold of toxicological concern (TTC) in risk assessment Toxicol Lett 2008 180 151 156 10.1016/j.toxlet.2008.05.006 18573621

[B28] Kristiansen E Madsen C Induction of protein droplet (alpha(2-mu)-globulin) nephropathy in male-rats after short-term dosage with 1,8-cineole and L-limonene Toxicol Lett 1995 80 147 152 10.1016/0378-4274(95)03390-7 7482582

